# Supplementary material for: Causal mediation and sensitivity analysis for mixed-scale data
Source: Stat Methods Med Res. 2023 May 17;32(7):1249–66. doi: 10.1177/09622802231173491 (PMC10500957; doi:10.1177/09622802231173491)
Supplement: sj-pdf-1-smm-10.1177_09622802231173491 - Supplemental material for Causal mediation and sensitivity analysis for mixed-scale data [file sj-pdf-1-smm-10.1177_09622802231173491.pdf]

Supplementary Material to Causal Mediation and Sensitivity Analysis for Mixed-Scale Data

Lexi Rene*, Antonio Linero†, and Elizabeth Slate‡

July 11, 2022

S.1 Algorithms

In this section we provide algorithms for average mediation effect estimation using the sensitivity analysis techniques developed in Section 5. Algorithm S.1 implements the logit-scale sensitivity analysis of Section 5.1 while Algorithm S.2 implements the linear-scale sensitivity analysis of Section S.2.

S.2 Sensitivity on the Linear Scale

Part of the motivation for introducing a sensitivity parameter through the logit link in Section 5.1 is that it is range preserving in the sense that it ensures that $0 < \mathbb{E}_\theta\{Y_i(a, m)\} < 1$. An alternative approach is to allow for possible violations of the range-preserving property and instead introduce λ through a linear link. We consider the following assumption to replace SI2:

*Department of Statistics, Florida State University, email: `lor16b@my.fsu.edu`
†Department of Statistics and Data Science, University of Texas at Austin, email: `antonio.linero@austin.utexas.edu`
‡Department of Statistics, Florida State University, email: `eslate@fsu.edu`

Algorithm S.1 Monte Carlo g-formula With Sensitivity Parameters

Input: $\theta, K, \rho, \lambda, \{X_i\}_{i=1}^N$

Sample $\omega \sim \text{Dirichlet}(1, \dots, 1)$

for $k = 1, \dots, K$ **do**

  for $i = 1, \dots, N$ **do**

    Sample $(U_0^\star, U_1^\star) \sim \text{Normal}(\mathbf{0}, \Sigma)$ where Σ is a correlation matrix with correlation ρ.

    Set $U_0 \leftarrow \Phi(U_0^\star)$ and $U_1 \leftarrow \Phi(U_1^\star)$

    for $a = 0, 1$ **do**

      Compute
      $\operatorname{logit} \alpha^M \leftarrow X_i^\top \beta_\alpha^M(a)$, $\operatorname{logit} \gamma^M \leftarrow X_i^\top \beta_\gamma^M(a)$,
      $\operatorname{logit} \mu^M \leftarrow X_i^\top \beta_\mu^M(a)$, $\log \phi^M \leftarrow X_i^\top \beta_\phi^M(a)$.

      Set

$$M_{ik}^\star(a) \leftarrow \begin{cases} 0 & \text{if } U_a < \alpha^M, \\ 1 & \text{if } U_a > 1 - (1 - \alpha^M)\gamma^M, \\ F_{\text{Beta}}^{-1}\{U_a' \mid \mu^M \phi^M, (1 - \mu^M)\phi^M\} & \text{otherwise} \end{cases}$$

      where $U_a' = (U_a - \alpha^M)/[(1 - \alpha^M)(1 - \gamma^M)]$.

      for $a' = 0, 1$ **do**

        Compute
        $\operatorname{logit} \alpha^Y \leftarrow (X_i, M_{ik}^\star(a))^\top \beta_\alpha^Y(a')$, $\operatorname{logit} \gamma^Y \leftarrow (X_i, M_{ik}^\star(a))^\top \beta_\gamma^Y(a')$,
        $\operatorname{logit} \mu^Y \leftarrow (X_i, M_{ik}^\star(a))^\top \beta_\mu^Y(a')$, $\log \phi^Y \leftarrow (X_i, M_{ik}^\star(a))^\top \beta_\phi^Y(a')$.

        Set $E \leftarrow (1 - \alpha^Y)\gamma^Y + (1 - \alpha^Y)(1 - \gamma^Y)\mu^Y$.

        Set $Y_{ik}^\star\{a', M_{ik}^\star(a)\} \leftarrow \operatorname{expit}\{\operatorname{logit} E + \lambda\{M_{ik}^\star(a') - M_{ik}^\star(a)\}$.

      end for

    end for

  end for

end for

Approximate $\delta(a), \zeta(a), \tau$ with

$$\delta(a) \approx K^{-1} \sum_{i,k} \omega_i [Y_{ik}^\star\{a, M_{ik}^\star(1)\} - Y_{ik}^\star\{a, M_{ik}^\star(0)\}]$$

$$\zeta(a) \approx K^{-1} \sum_{i,k} \omega_i [Y_{ik}^\star\{1, M_{ik}^\star(a)\} - Y_{ik}^\star\{0, M_{ik}^\star(a)\}]$$

$$\tau \approx K^{-1} \sum_{i,k} \omega_i [Y_{ik}^\star\{1, M_{ik}^\star(1)\} - Y_{ik}^\star\{0, M_{ik}^\star(0)\}]$$

for $a = 0, 1$

return $\{\delta(0), \delta(1), \zeta(0), \zeta(1)\}$

SI2C Conditional on X_i, $M_i(0)$, and $M_i(1)$, the mean of $Y_i(a, m)$ is given by

$$\mathbb{E}_\theta\{Y_i(a,m) \mid M_i(a), M_i(a'), X_i\} = r_y(m,a,x) + \lambda\{M_i(a) - m\}$$

where $r_y(m,a,x) = \mathbb{E}_\theta(Y_i \mid M_i = m, A_i = a, X_i = x)$.

The following result establishes that SI2C identifies the average causal mediation effects.

Proposition 2. *Suppose that SI1, SI2C, and SI3 hold. Then we have*

$$\mathbb{E}_\theta[Y_i\{a, M_i(a')\}] = \iint r_y(m,a,x)\, f_\theta(M_i = m \mid A_i = a', X_i = x)\ dm\ F_X(dx) + \lambda[\mathbb{E}_\theta\{M_i(a)\} - \mathbb{E}_\theta\{M_i(a')\}].$$

Proof. As in the proof of Proposition 1, iterated expectation gives

$$\mathbb{E}_\theta[Y_i\{a, M_i(a')\}] = \mathbb{E}_\theta[r_y\{M_i(a'), a, X_i\} + \lambda\{M_i(a) - M_i(a')\}], \tag{S.9}$$

so that $\mathbb{E}_\theta[Y_i\{a, M_i(a')\}] = \mathbb{E}_\theta[r_y\{M_i(a'), a, X_i\}] + \lambda[\mathbb{E}_\theta\{M_i(a)\} - \mathbb{E}_\theta\{M_i(a')\}]$. An application of SI1 gives

$$\begin{aligned}
\mathbb{E}_\theta[r_y\{M_i(a'), a, X_i\}] &= \iint r_y(m,a,x)\, f_\theta\{M_i(a') = m \mid X_i = x\}\ dm\ F_X(dx) \\
&= \int r_y(m,a,x)\, f_\theta\{M_i(a') = m \mid A_i = a', X_i = x\}\ dm\ F_X(dx) \\
&= \iint r_y(m,a,x)\, f_\theta(M_i = m \mid A_i = a', X_i = x)\ dm\ F_X(dx).
\end{aligned}$$

Plugging this expressions for $\mathbb{E}_\theta[r_y\{M_i(a'), a, X_i\}]$ into (S.9) finishes the proof. □

SI2C has several advantages over SI2A — SI2B. First, we feel that shifts directly on the scale of the mean are more easily interpreted than shifts on the logit scale. Like the shift on the logit scale developed in Section 5.1, λ can be interpreted as shifting the causal effect of m into an association with $M_i(a)$. For example, if we had used the linear model

$r_y(m, a, x) = \beta_0^Y + x^\top \beta_X^Y + a\,\beta_A^Y + m\,\beta_M^Y$ then we could rewrite $\mathbb{E}_\theta\{Y_i(a, m) \mid M_i(a), M_i(a'), X_i\}$ as $\beta_0^Y + x^\top \beta_X^Y + a\,\beta_A^Y + m\,(\beta_M^Y - \lambda) + M_i(a)\,\lambda$. To a elicit a default range of λ's we can use the approach outlined in Section 5.1 by fitting a linear regression of Y_i on (M_i, A_i, X_i).

The second benefit of SI2C is that the linearity removes the need to specify the correlation ρ between $M_i(a)$ and $M_i(a')$ so that the sensitivity analysis only requires eliciting a range of plausible λ's. This is very helpful, as ρ is more difficult to interpret than λ.

Using Proposition 2 we can again develop a Monte Carlo implementation of the g-formula using the approximation $\mathbb{E}_\theta[Y_i\{a, M_i(a')\}] \approx K^{-1} \sum_{i,k} \omega_i \, Y_{ik}^\star\{a, M_{ik}^\star(a')\},]$ where $Y_{ik}^\star\{a, M_{ik}^\star(a')\} = r_y\{M_{ik}^\star(a'), a, X_i\} + \lambda\{M_{ik}^\star(a) - M_{ik}^\star(a')\}$ and $M_{ik}^\star(a)$ is sampled from $f_\theta(M_i \mid A_i = a, X_i)$. A possible algorithm is given in Algorithm S.2.

Figure S.1 gives a sensitivity analysis under SI2C for the JOBS II data, with a reasonable range for λ now obtained from a linear regression of Y_i on (A_i, M_i, X_i). The results are substantively in agreement with the logit-scaled sensitivity analysis: there are no values of λ that lead to evidence of either direct or indirect effects of the treatment.

For the linear model, it is easy to see why λ does not greatly influence our conclusions: as shown in Proposition 2, the influence of λ is through the average treatment effect on the mediator $\varpi = \mathbb{E}_\theta(M_i \mid A_i = 1) - \mathbb{E}_\theta(M_i \mid A_i = 0)$. Because the sign of ϖ is uncertain, the direction by which λ shifts $\mathbb{E}_\theta[Y_i\{a, M_i(a')\}]$ is itself uncertain, so that increasing $|\lambda|$ has more effect on the uncertainty of the mediation effects than it does on the point estimates.

S.3 Sensitivity Analysis for Different Correlations

Figure S.2 and Figure S.3 give the sensitivity analyses for the values of $\rho = 0$ and $\rho = 0.5$, respectively, under SI2B.

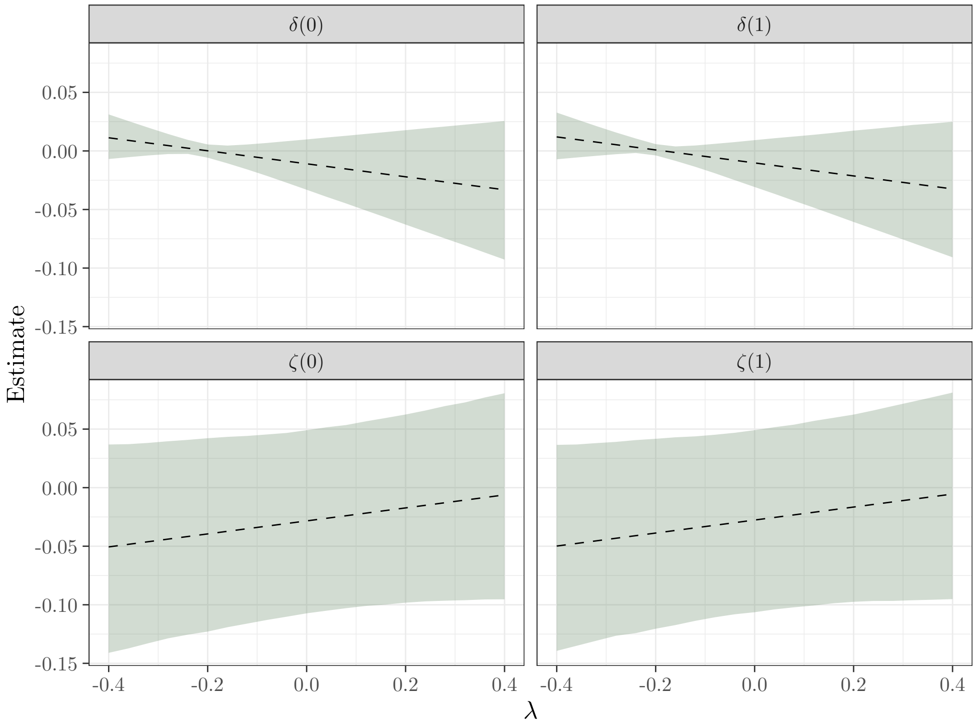

Figure S.1: Sensitivity of inferences about $\delta(a)$ and $\zeta(a)$ to changes in the sensitivity parameter λ under assumption SI2C. The dashed line is the posterior mean, and the bands delimit a pointwise 95% credible interval.

S.4 MCMC Diagnostics

Figure S.4 shows traceplots to assess mixing of the Markov chains used to fit the JOBS data on the causal mediation effects. Figure S.5 shows the same for the log posterior density. In both cases, we see that the Markov chain is mixing rapidly, and there is no evidence of any problems.

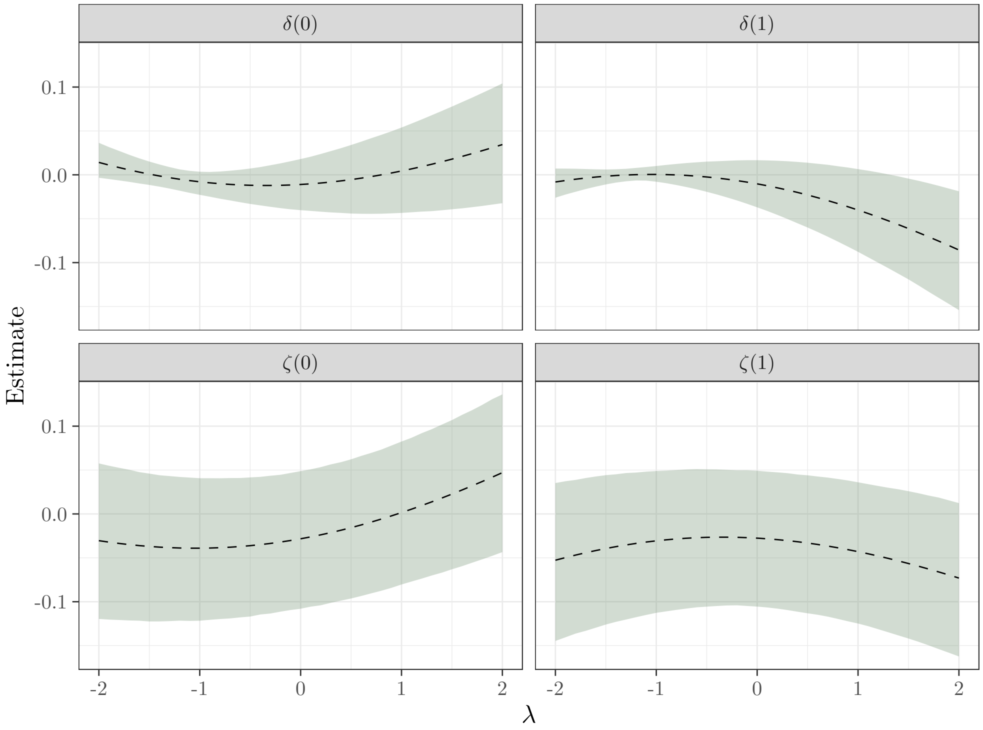

Figure S.2: Sensitivity of inferences about $\delta(a)$ and $\zeta(a)$ to changes in the sensitivity parameter λ under assumptions SI2A and SI2B when $\rho = 0$. The dashed line is the posterior mean, and the bands delimit a pointwise 95% credible interval.

Algorithm S.2 Monte Carlo g-formula With Linear Sensitivity Parameters

Input: $\theta, K, \lambda, \{X_i\}_{i=1}^N$

Sample $\omega \sim \text{Dirichlet}(1, \ldots, 1)$
for $k = 1, \ldots, K$ **do**
  for $i = 1, \ldots, N$ **do**
    Sample $U \sim \text{Uniform}(0, 1)$
    for $a = 0, 1$ **do**
      Compute
      $\operatorname{logit} \alpha^M \leftarrow X_i^\top \beta_\alpha^M(a)$, $\operatorname{logit} \gamma^M \leftarrow X_i^\top \beta_\gamma^M(a)$,
      $\operatorname{logit} \mu^M \leftarrow X_i^\top \beta_\mu^M(a)$, $\log \phi^M \leftarrow X_i^\top \beta_\phi^M(a)$.
      Set

$$M_{ik}^\star(a) \leftarrow \begin{cases} 0 & \text{if } U < \alpha^M, \\ 1 & \text{if } U > 1 - (1-\alpha^M)\gamma^M, \\ F_{\text{Beta}}^{-1}\{U' \mid \mu^M \phi^M, (1-\mu^M)\phi^M\} & \text{otherwise} \end{cases}$$

      where $U' = (U - \alpha^M)/[(1-\alpha^M)(1-\gamma^M)]$.
      for $a' = 0, 1$ **do**
        Compute
        $\operatorname{logit} \alpha^Y \leftarrow (X_i, M_{ik}^\star(a))^\top \beta_\alpha^Y(a')$, $\operatorname{logit} \gamma^Y \leftarrow (X_i, M_{ik}^\star(a))^\top \beta_\gamma^Y(a')$,
        $\operatorname{logit} \mu^Y \leftarrow (X_i, M_{ik}^\star(a))^\top \beta_\mu^Y(a')$, $\log \phi^Y \leftarrow (X_i, M_{ik}^\star(a))^\top \beta_\phi^Y(a')$.
        Set $Y_{ik}^\star\{a', M_i(a)\} \leftarrow (1-\alpha^Y)\gamma^Y + (1-\alpha^Y)(1-\gamma^Y)\mu^Y + \lambda\{M_{ik}^\star(a') - M_{ik}^\star(a)\}$.
      end for
    end for
  end for
end for
Approximate $\delta(a), \zeta(a), \tau$ with

$$\delta(a) \approx K^{-1} \sum_{i,k} \omega_i [Y_{ik}^\star\{a, M_{ik}^\star(1)\} - Y_{ik}^\star\{a, M_{ik}^\star(0)\}]$$

$$\zeta(a) \approx K^{-1} \sum_{i,k} \omega_i [Y_{ik}^\star\{1, M_{ik}^\star(a)\} - Y_{ik}^\star\{0, M_{ik}^\star(a)\}]$$

$$\tau \approx K^{-1} \sum_{i,k} \omega_i [Y_{ik}^\star\{1, M_{ik}^\star(1)\} - Y_{ik}^\star\{0, M_{ik}^\star(0)\}]$$

for $a = 0, 1$
return $\{\delta(0), \delta(1), \zeta(0), \zeta(1)\}$

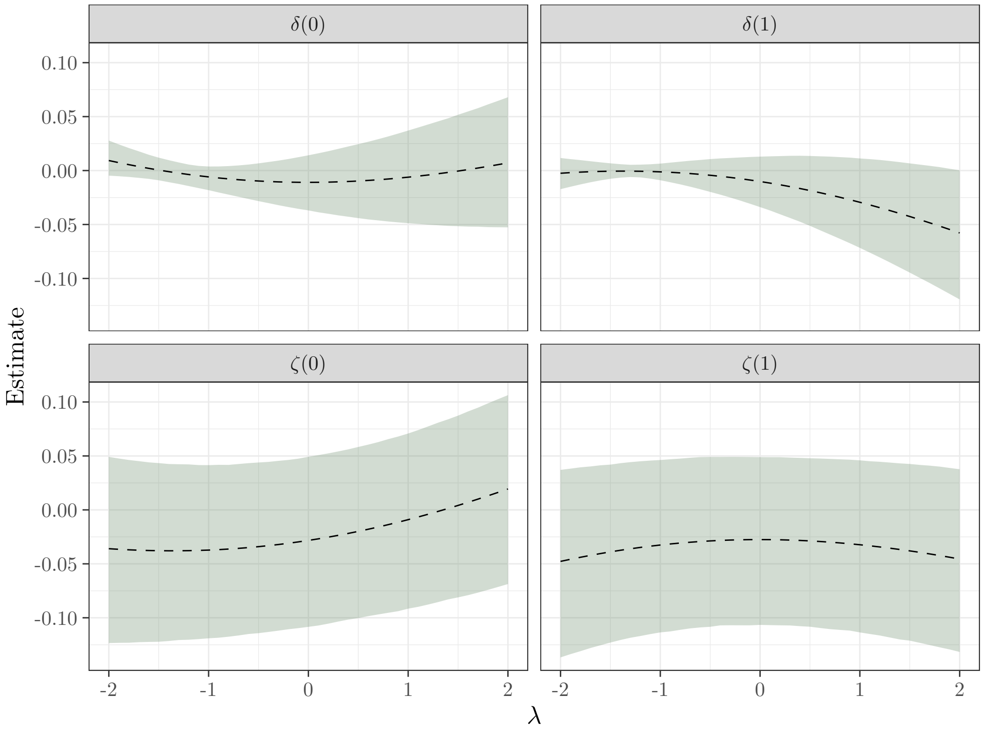

Figure S.3: Sensitivity of inferences about $\delta(a)$ and $\zeta(a)$ to changes in the sensitivity parameter λ under assumptions SI2A and SI2B when $\rho = 0.5$. The dashed line is the posterior mean, and the bands delimit a pointwise 95% credible interval.

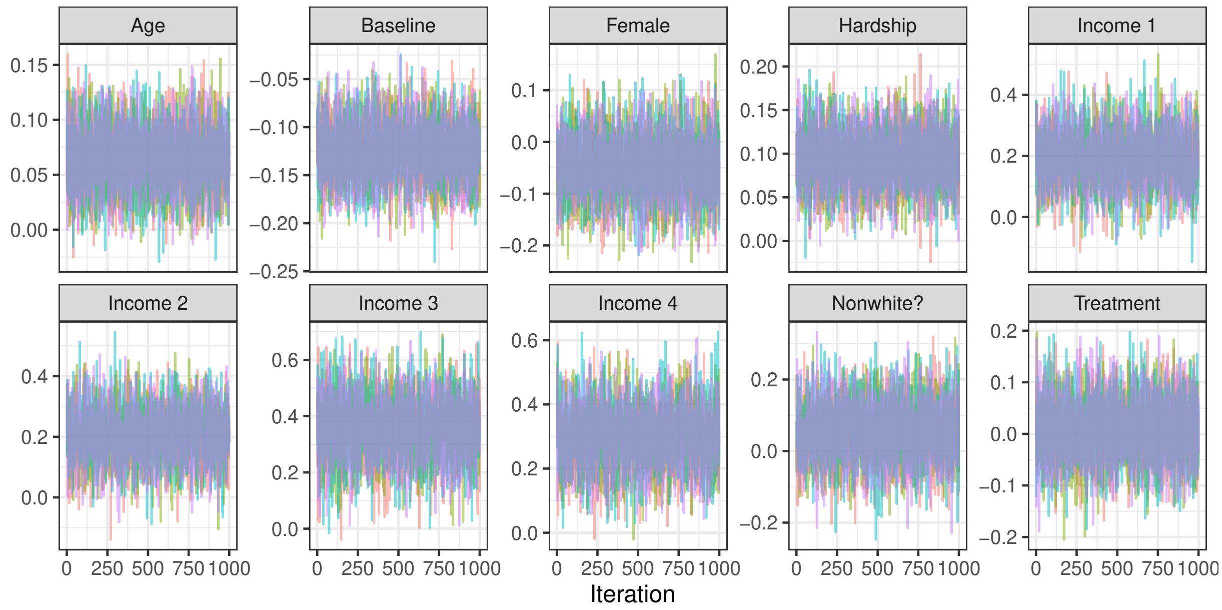

Figure S.4: Mixing of our four Markov chains for the regression coefficients β_μ^M after discarding the first 1000 iterations to burn-in. Results are similar for the other sets of regression coefficients. Baseline denotes the baseline depression level, Hardship denotes the numeric measure of economic hardship, and Income 1 — Income 4 denote indicator variables for different income levels.

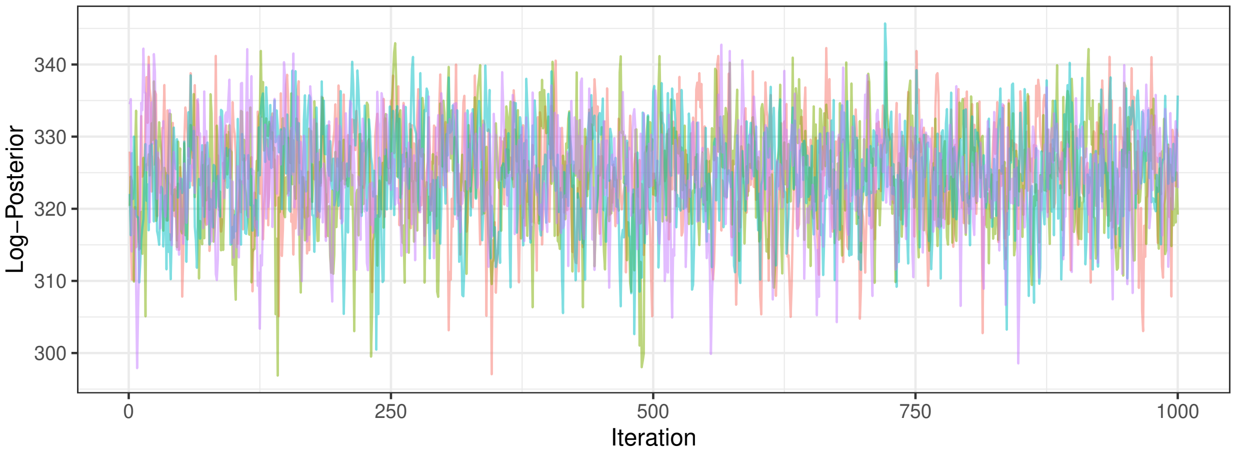

Figure S.5: Mixing of the log-posterior density across the four chains.
